# Gypensapogenin I Ameliorates Isoproterenol (ISO)-Induced Myocardial Damage through Regulating the TLR4/NF-κB/NLRP3 Pathway

**DOI:** 10.3390/molecules27165298

**Published:** 2022-08-19

**Authors:** Mengyuan Li, Hongyan Tan, Ting Gao, Linlin Han, Xinhang Teng, Fang Wang, Xiaoshu Zhang

**Affiliations:** School of Functional Food and Wine, Shenyang Pharmaceutical University, Shenyang 110016, China

**Keywords:** Jiaogulan tea, Gypensapogenin I, myocardiocytes, myocardial fibrosis

## Abstract

Myocardial fibrosis (MF) is a common pathological feature of many heart diseases and seriously threatens the normal activity of the heart. Jiaogulan (*Gynostemma pentaphyllum*) tea is a functional food that is commercially available worldwide. Gypensapogenin I (Gyp I), which is a novel dammarane-type saponin, was obtained from the hydrolysates of total gypenosides. It has been reported to exert a beneficial anti-inflammatory effect. In our study, we attempted to investigate the efficiency and possible molecular mechanism of Gyp I in cardiac injury treatment induced by ISO. In vitro, Gyp I was found to increase the survival rate of H9c2 cells and inhibit apoptosis. Combined with molecular docking and Western blot analysis, Gyp I was confirmed to regulate the TLR4/NF-κB/NLRP3 signaling pathway. In vivo, C57BL6 mice were subcutaneously injected with 10 mg/kg ISO to induce heart failure. Mice were given a gavage of Gyp I (10, 20, or 40 mg/kg/d for three weeks). Pathological alterations, fibrosis-, inflammation-, and apoptosis-related molecules were examined. By means of cardiac function detection, biochemical index analysis, QRT-PCR monitoring, histopathological staining, immunohistochemistry, and Western blot analysis, it was elucidated that Gyp I could improve cardiac dysfunction, alleviate collagen deposition, and reduce myocardial fibrosis (MF). In summary, we reported for the first time that Gyp I showed good myocardial protective activity in vitro and in vivo, and its mechanism was related to the TLR4/NF-κB/NLRP3 signaling pathway.

## 1. Introduction

The heart is one of the first organs to develop throughout embryogenesis [1]. Dynamic phenotypic changes in capillary endothelial cells and cardiac fibroblasts support the heart’s normal contractile function [2]. Many heart diseases are associated with fibrosis. Myocardial fibrosis (MF) is a common pathological feature of many end-stage cardiovascular diseases and is highly associated with cellular events, including cardiac myocyte disorder, recruitment of inflammatory cells, deposition of extracellular matrix components, proliferation of myocardial interstitial fibroblasts, and impairment of normal cardiac contractile function [3,4,5]. Fibrosis can manifest in two types, reactive gap fibrosis or replacement fibrosis. In reactive gap fibrosis, the myocardial collagen fibers are abnormally increased and deposited in the myocardial interstitium to form scarring and chronic expansion, without obvious myocardial cell loss and necrosis. On the other hand, replacement fibrosis is a repair process in which collagen secreted by dead cells replaces the cardiomyocytes within the cavity [6,7]. In general, MF occurs when the heart is unable to maintain normal pressure resulting in overload or a weakened adaptive response after myocardial cell damage [8]. The persistent accumulation of long-term fibrotic proteins can result in irreversible effects, thereby leading to permanent ventricular remodeling and severe impairment of cardiac function, gradually developing into heart failure [9].

The impact of fibrogenesis on impaired cardiac function has been increasingly recognized. Isoproterenol (ISO), a synthetic β-adrenergic receptor agonist, causes myocardial damage and induces chronic inflammatory responses in the heart with long-term effects [10]. The pathogenesis of MF is closely related to chronic inflammatory responses [6]. When myocardial tissue is damaged, the overexpression of oxidative active substances in the body causes inflammatory infiltration of neutrophils that release a large number of inflammatory factors, such as IL-6, IL-8 and TNF-α, resulting in abnormal accumulation of collagen fibers [11,12]. In addition, a series of biological processes, such as cardiomyocyte hypertrophy, embryonic gene expression, and cardiomyocyte apoptosis, are involved in the development of MF [13].

Jiaogulan (*Gynostemma pentaphyllum* (Thunb.) Makino) has been used as an edible herbal folk medicine as early as the Ming Dynasty in China, with a stable medicinal and mild effect [14,15]. Saponins isolated from Jiaogulan have a dammarane-type saponin structure that is similar to that of ginsenosides and are considered bioactive components with various pharmacological properties, including anti-inflammatory effects [16], antioxidative effects [17], and arteriosclerosis prevention [18], and they are also used for the treatment of diabetes [19]. Gypensapogenin I (Gyp I), a secondary product obtained from the acid hydrolysis of gypenosides, has a tetracyclic triterpene dammarane skeleton structure [20], and the mechanism underlying the efficacy of Gyp I on cardio-protection is still unclear.

Toll-like receptors are a class of pathogen pattern recognition receptors associated with innate immune diseases [21,22]. Among them, TLR4 is widely present in the cardiovascular system and is mainly responsible for mediating inflammatory responses initiated by endotoxin and lipopolysaccharide. The TLR4 response is closely related to the occurrence, development, and prognosis of cardiovascular diseases [23,24]. TLR4 is highly expressed in many cardiovascular diseases. It induces nuclear expression of NF-κB by triggering inflammatory signaling cascades and specifically binds to a variety of proinflammatory cytokines to induce MF [25].

To maintain normal function and structure, the cardiovascular system requires a balance of cell formation and death [26]. Pyroptosis is a type of proinflammatory programmed cell death associated with a variety of diseases. When cells are stimulated by infectious or endogenous injury-related signals, inflammasomes mediate the process of pyroptosis, leading to the formation of pore membranes in the plasma membrane and within cells. Simultaneously, cells will swell and rupture, resulting in the extravasation of massive cytoplasmic inflammatory cytokine content [27,28]. The pyrin domain of NOD-, LRR-, and pyrin domain-containing protein 3 (NLRP3) is the most direct pivotal link between the regulation of pyroptosis and the inflammatory response and is of great significance in mediating pyroptosis and inducing MF [29]. When NF-κB enters the nucleus and initiates transcription of the NLRP3 gene, it recruits apoptosis-associated speck-like protein and pro-caspase-1 to form the NLRP3-ASC-procaspase-1 inflammasome complex, which in turn stimulates the inflammasome that triggers the activation of caspase-1 and promotes the cleavage, processing, and secretion of the proinflammatory cytokines IL-1β and IL-18 [30,31]. Gasdermin D (GSDMD) is cleaved by caspase-1 to form an N-terminal fragment and translocates to the cell membrane for oligomerization to form GSDMD pores that can release mature IL-1β, IL-18, and other inflammatory factors and subsequently release cytoplasmic substances, thereby aggravating the inflammatory response [28,32,33].

Apoptosis is a programmed death mode that is different from the pyroptotic effect [34]. Several studies have shown that the inflammasome-mediated pyroptosis pathway can directly trigger the apoptotic pathway [35,36]. The inflammasome triggers the activation of the caspase network, which promotes pyroptosis and apoptotic cell death. In cells in which rapid pyroptosis is blocked, caspase-1 may still undergo delayed inflammasome-dependent apoptosis [37], whereas GSDMD-N penetrates the mitochondria and induces the inflammasome to activate downstream apoptotic bodies, thereby enhancing the mitochondrial apoptotic pathway [38]. In the present study, we deployed an ISO-induced myocardial injury model to investigate the protective effect of Gyp I on the heart and its underlying mechanism involved, both in vitro and in vivo.

## 2. Results

### 2.1. Gyp I Improves the ISO-Attenuated H9c2 Cell Viability

**Figure 1:**
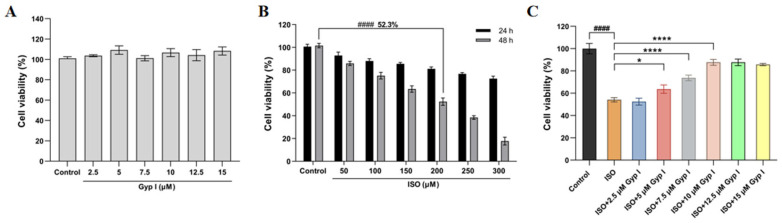
MTT assay to detect the survival rate of H9c2 cardiomyocytes: (**A**) Effect of Gyp I (2.5–15 μM) on the viability of H9c2 cells at 48 h. (**B**) Effects of ISO (50–300 μM) on H9c2 cell viability at 24 and 48 h. (**C**) Effects of Gyp I (5–15 μM) on the viability of H9c2 cardiomyocytes induced by ISO (200 μM). Results are presented as mean ± SD of three independent experiments using one-way ANOVA followed by Tukey’s multiple-comparison test (####: *p* < 0.0001 compared with Control group; *: *p* < 0.05, ****: *p* < 0.0001 compared with ISO group).

The effect of Gyp I on the viability of H9c2 cells was determined using the methyl thiazole tetrazolium (MTT) assay. The results showed that Gyp I (2.5–15 μM) did not affect the growth and viability of H9c2 cells and was nontoxic after a 48 h incubation period for the Control group (Figure 1A). Furthermore, ISO (50–300 μM) significantly reduced cardiomyocyte viability at 48 h instead of 24 h. In addition, ISO (200 μM) had a cell viability rate of 52.3%, which was suitable for the subsequent optimal modeling concentration (Figure 1B). To further investigate the effect of Gyp I on ISO-induced cardiotoxicity, H9c2 cells were first preincubated with Gyp I (0–15 μM) for 4 h, and ISO (200 μM) was then added, and cells were co-incubated with Gyp I for 48 h. The results showed that Gyp I treatment (5–15 μM) enhanced the viability of H9c2 cells and restored ISO-induced cardiotoxic effects (Figure 1C).

### 2.2. Gyp I Inhibits ISO-Induced Apoptosis of H9c2 Cardiomyocytes

**Figure 2:**
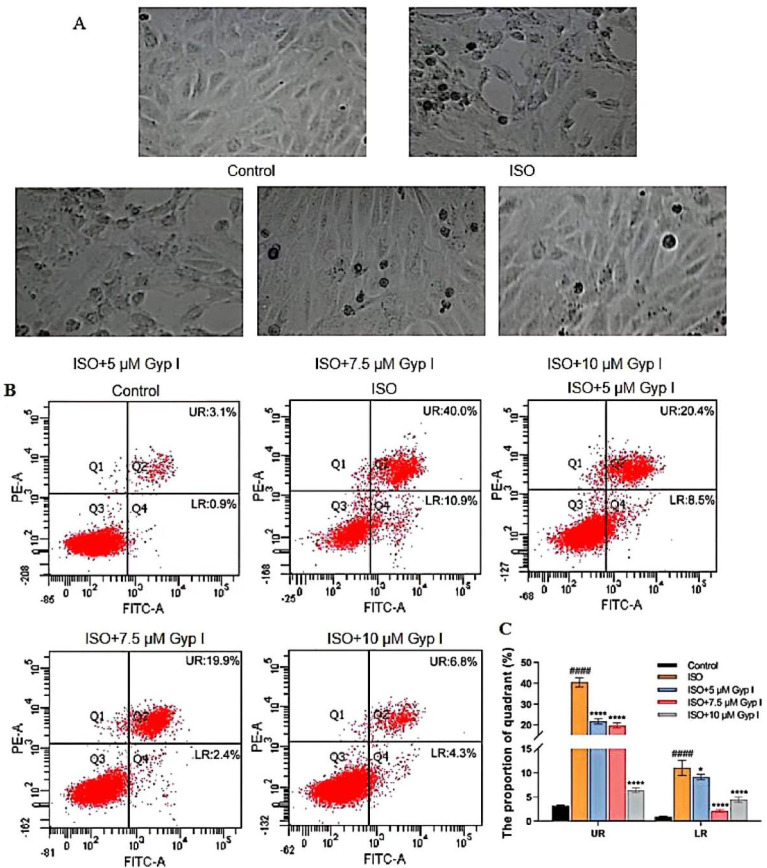
Gyp I protects ISO-induced apoptosis in H9c2 cells: (**A**) Morphology of Gyp I on ISO-induced apoptosis in H9c2 cells (40×). (**B**) Analysis of apoptosis using the Annexin/Propidium lodide (PI) assay of H9c2 cells in the Control group, ISO group, and Gyp I (5, 7.5, and 10 μM) groups. (**C**) Histogram of Gyp I on the degree of early and late apoptosis of H9c2 cells induced by ISO (200 μM). Results are presented as mean ± SD of three independent experiments using one-way ANOVA followed by Tukey’s multiple-comparison test (####: *p* < 0.0001 compared with Control group; *: *p* < 0.05, ****: *p* < 0.0001 compared with ISO group).

As shown in Figure 2A, compared with the Control group, the cell morphology of the ISO group was significantly damaged and deformed, accompanied by a large number of apoptotic bodies. The Gyp I-treated groups (5, 7.5, and 10 μM) significantly reduced ISO-induced apoptosis of H9c2 cells in a dose-dependent manner, and the cell morphology gradually returned to normal. To further explore the apoptosis inhibitory effect of Gyp I on ISO-induced cardiomyocyte injury, H9c2 cells were analyzed by flow cytometry. The upper right (UR) and lower right (LR) quadrants represent late and early apoptotic cells, respectively. The results indicated that Gyp I significantly attenuated ISO-induced apoptosis in a dose-dependent manner. Importantly, the Gyp I-treated group (10 μM) showed the best preventive effect; the ratio of UR and LR apoptotic cells were 6.8% and 4.3%, respectively (Figure 2B,C).

### 2.3. Gyp I Alters Expression of Apoptosis-Related Proteins in ISO-Treated H9c2 Cells

**Figure 3:**
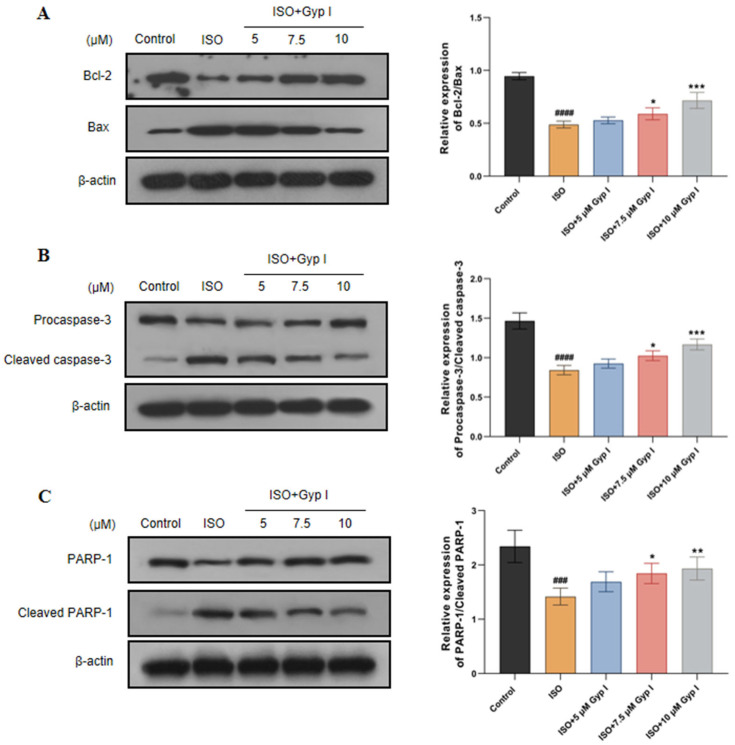
Effects of Gyp I on the expression of apoptosis-related proteins in ISO-induced H9c2 cells were analyzed by Western blot: (**A**) Protein expression assay of Bcl-2 and Bax. (**B**) Protein expression assay of Procaspase-3 and Cleaved caspase-3. (**C**) Protein expression assay of PARP-1 and Cleaved PARP-1. Results are presented as mean ± SD of three independent experiments using one-way ANOVA followed by Tukey’s multiple-comparison test (###: *p* < 0.001, ####: *p* < 0.0001 compared with Control group; *: *p* < 0.05, **: *p* < 0.01, ***: *p* < 0.001 compared with ISO group).

To further understand the mechanism of Gyp I protection regarding the regulation of apoptosis, we measured the effect of Gyp I on apoptosis-related protein expression using Western blot analysis. As shown in Figure 3A, compared with the Control group, the expression of the anti-apoptotic protein Bcl-2 was decreased in the ISO group, while the pro-apoptotic protein Bax was increased. Conversely, Gyp I dose-dependently increased Bcl-2/Bax expression compared with the ISO group. ISO-induced injury of H9c2 cells markedly reduced Procaspase-3 and PARP-1 expression, which resulted in caspase-3 activation and PARP-1 cleavage. However, Gyp I increased Procaspase-3/Cleaved caspase-3 and PARP-1/Cleaved PARP-1 expression ratios in a dose-dependent manner compared with ISO groups (Figure 3B,C). These findings suggest that Gyp I significantly inhibited ISO-induced expression of apoptosis-related proteins in H9c2 cells.

### 2.4. Gyp I Blocks Activation of the ISO-Induced TLR4/NF-κB/NLRP3 Signaling Pathway

**Figure 4:**
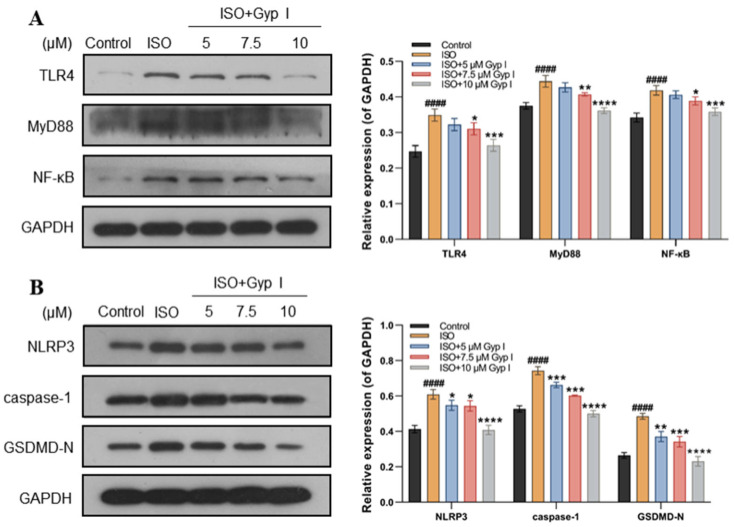
Effect of Gyp I on the expression of the TLR4/NF-κB/NLRP3 signaling pathway in vitro: (**A**) The relative expression of TLR4, MyD88, and NF-κB. (**B**) The relative expression of NLRP3, caspase-1, and GSDMD-N. Results are presented as mean ± SD of three independent experiments using one-way ANOVA followed by Tukey’s multiple-comparison test (####: *p* < 0.0001 compared with Control group; *: *p* < 0.05, **: *p* < 0.01, ***: *p* < 0.001, ****: *p* < 0.0001 compared with ISO group).

To explore the protective mechanism of Gyp I on ISO-induced H9c2 cell injury, we evaluated whether Gyp I blocks the ISO-induced activation of the TLR4/NF-κB/NLRP3 signaling pathway in H9c2 cardiomyocytes. The results indicated that the expression of TLR4, downstream protein MyD88, and NF-κB in cardiomyocytes treated with ISO was significantly increased when compared with those of Gyp I-treated groups (Figure 4A). In addition, the protein expression of NLRP3, caspase-1, and the key factor of pyroptosis, GSDMD-N, was significantly increased in the ISO group. It is worth noting that, compared with the ISO group, the protein expressions of NLRP3, caspase-1, and GSDMD-N in the Gyp-treated groups were significantly decreased, thereby indicating that Gyp I inhibited NLRP3 and the downstream pyroptosis expression of related proteins (Figure 4B).

### 2.5. Effects of Gyp I on Cardiac Morphology and Weight in ISO-Treated Mice

**Figure 5:**
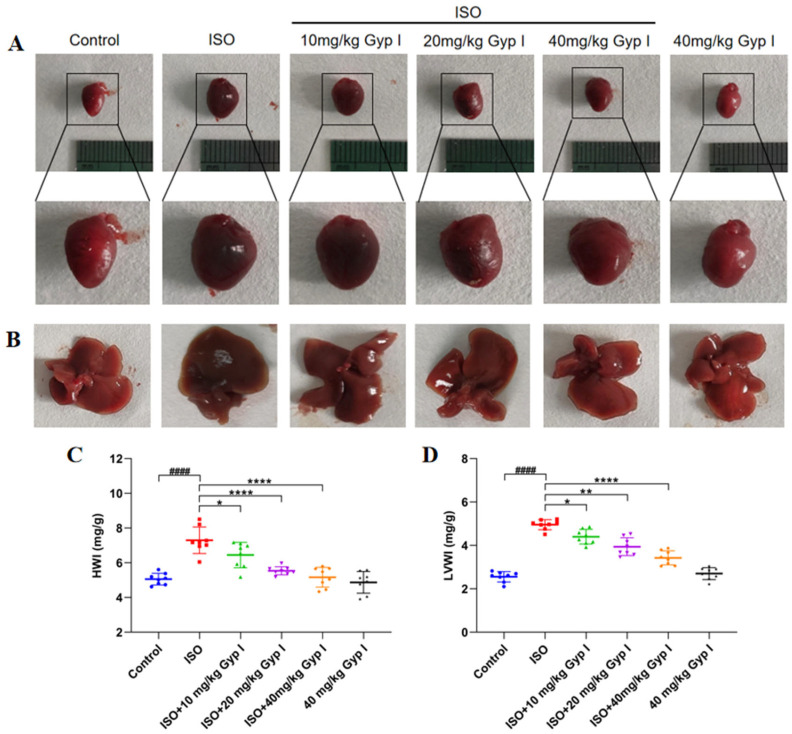
Effects of Gyp I on heart and liver morphology; HWI and LVWI of ISO-induced mice: (**A**) Cardiac morphology. (**B**) Liver morphology. (**C**) HWI. (**D**) LVWI. Analysis was conducted using one-way ANOVA followed by Tukey’s multiple-comparison test (*n* = 8, mean ± SD, ####: *p* < 0.0001 compared with Control group; *: *p* < 0.05, **: *p* < 0.01, ****: *p* < 0.0001 compared with ISO group).

As shown in Figure 5A, the normal heart has a moderately inverted cone in shape with a bright red and smooth surface. In contrast, the volume of the ISO-treated heart was significantly increased, with thickened ventricular walls and an overall dark red appearance. After Gyp I (10, 20, and 40 mg/kg) intervention, the heart volume returned to normal, and the color gradually changed to bright red in a dose-dependent manner. We observed the liver morphology and found that the liver surface in the ISO group was rough, and the liver was dull in color. However, the liver morphology of the Gyp I intervention group was gradually improved with an increased dose, and it was similar to that of the Control group at the highest dose concentration (Figure 5B). Furthermore, compared with the Control group, the heart and liver of the group treated with Gyp I (40 mg/kg) alone (non- ISO induced) did not show obvious changes, which indicated that Gyp I had no toxic effect on mice. The effects of Gyp I were explored on the whole heart and the left ventricular weights of ISO-induced mice hearts, and it was found that the heart weight index (HWI) index of the ISO group was significantly increased, while the HWI level of the Gyp I intervention groups was opposite to that of the ISO group (Figure 5C). The left ventricular weight index (LVWI) results were also similar, as shown in Figure 5D.

### 2.6. Electrocardiogram (ECG) Analysis of Various Treatment Group Mice

**Figure 6:**
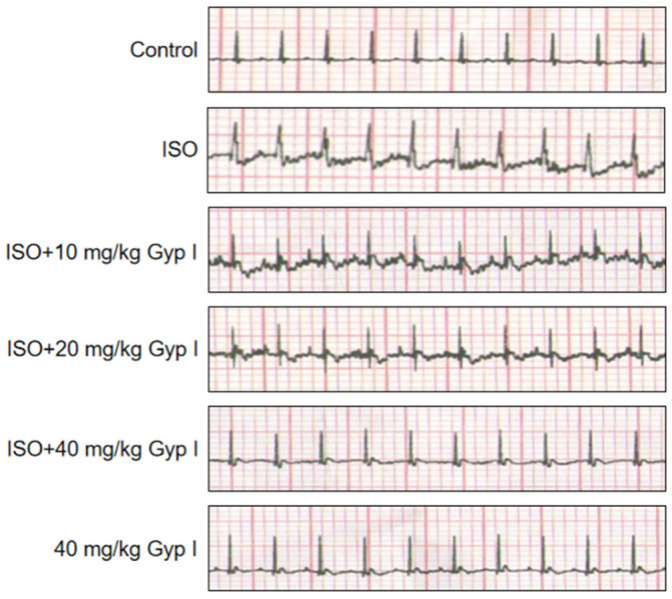
Representative images of ECG analysis of various treatment group mice.

The effect of Gyp I on electrical activity was determined for the hearts of mice in all groups and was measured by electrocardiographic patterns. Figure 6 shows that ISO-treated mice exhibited depressed ST-segment, T-wave depression, and impaired myocardial contractility, in comparison to the normal ECG pattern observed in untreated mice. After Gyp I (10 and 20 mg/kg) intervention, mild ST-segment elevation and T-wave inversion were observed, respectively, which returned to normal ECG pattern with Gyp I (40 mg/kg). Surprisingly, the group treated with Gyp I alone showed an almost normal ECG pattern throughout the study period. Thus, the above finding indicated that ISO-induced cardiac function injury was gradually protected by Gyp I in a dose-dependent manner.

### 2.7. Effect of Gyp I on ISO-Induced Oxidative Stress in Mice Hearts

**Figure 7:**
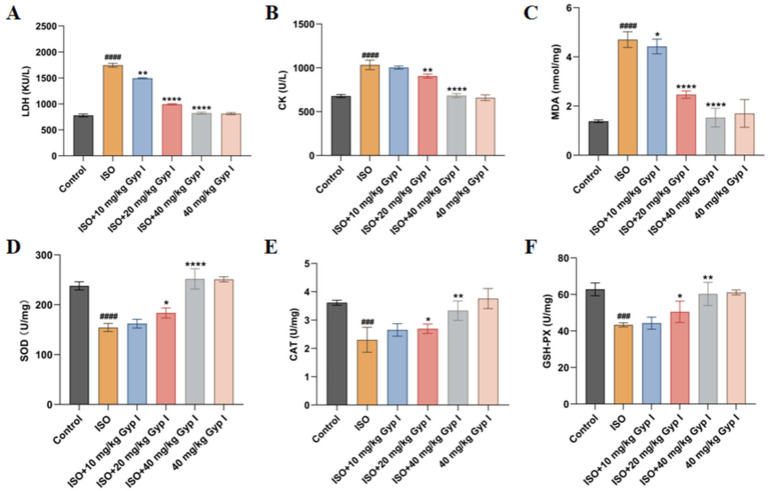
Biochemical assessment in serum and tissues of mice in each group: (**A**) LDH levels in serum. (**B**) CK levels in serum. (**C**) MDA levels in tissues. (**D**) SOD levels in tissues. (**E**) CAT levels in tissues. (**F**) GSH-PX levels in tissues (*n* = 5, mean ± SD, ###: *p* < 0.001, ####: *p* < 0.0001 compared with Control group; *: *p* < 0.05, **: *p* < 0.01, ****: *p* < 0.0001 compared with ISO group).

The level of oxidative stress was changed in the hearts of ISO-induced mice, thereby promoting the production of reactive oxygen species (ROS) and impairing the normal work of the heart. To evaluate the effects of Gyp I on the levels of biomarker enzymes, biochemical analysis was performed as shown in Figure 7. Compared with the Control group, the serum levels of lactate dehydrogenase (LDH) and creatine kinase (CK) of the ISO group were drastically increased. The ISO + Gyp I intervention groups (10, 20, and 40 mg/kg) showed a significantly reduced content compared to the ISO group (Figure 7A,B). Additionally, we found consistent results among malondialdehyde (MDA) with the above two enzymes in tissue samples (Figure 7C). Superoxide Dismutase (SOD), catalase (CAT), and glutathione peroxidase (GSH-PX), important antioxidant factors, were significantly reduced in tissue samples from mice treated with ISO. However, the concomitant treatment with Gyp I elicited a dose-dependent increase in levels at low, medium, and high doses (Figure 7D–F). Surprisingly, Gyp I (40 mg/kg) treatment alone had no effect on the level of oxidative stress in serum and tissues.

### 2.8. Effect of Gyp I on the mRNA Expression of ISO-Induced Myocardial Hypertrophy Genes in Mice

**Figure 8:**
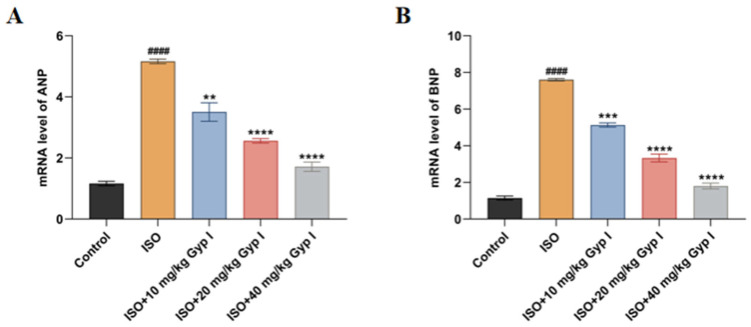
Effects of Gyp I on ISO-induced myocardial hypertrophy mRNA levels in mice: (**A**) ANP. (**B**) BNP (*n* = 3, mean ± SD, ####: *p* < 0.0001 compared with Control group; **: *p* < 0.01, ***: *p* < 0.001, ****: *p* < 0.0001 compared with ISO group).

To determine whether Gyp I interfered with ISO-induced changes in the levels of cardiac embryonic genes, the mRNA expression levels of the hypertrophic markers atrial natriuretic peptide (ANP) and brain natriuretic peptide (BNP) in myocardial tissue were detected by real-time quantitative PCR. The primer sequences are shown in Table 1. Compared with the Control group, the expression levels of the two markers in the ISO model group were significantly increased, while the two markers of ISO groups treated concomitantly with Gyp I decreased in a dose-dependent manner (Figure 8).

### 2.9. Gyp I Interferes with ISO-Induced MF in Mice

**Figure 9:**
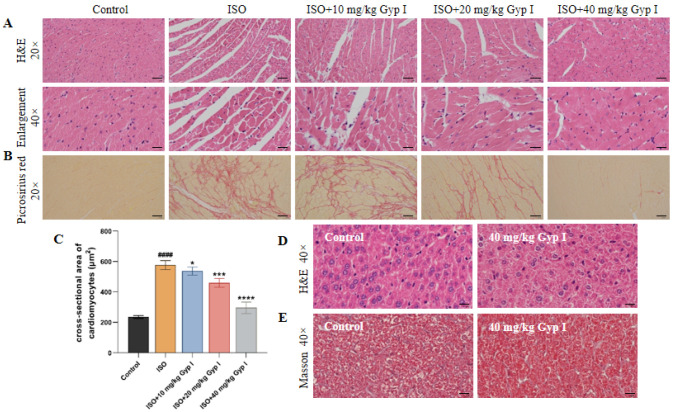
Representative images of histopathological studies of heart and liver sections: (**A**) H&E staining of heart sections. (**B**) Sirius red staining of heart sections. (**C**) Cardiomyocytes cross-sectional area (μm^2^) in tissue after H&E staining. (**D**) Liver section H&E staining. (**E**) Masson staining of liver section (Enlargement: 20×, Scale bar: 100 μm; Enlargement: 40×, Scale bar: 50 μm; *n* = 3, mean ± SD, #### *p* < 0.0001 compared with Control group; * *p* < 0.05, *** *p* < 0.001, **** *p* < 0.0001 compared with ISO group).

To determine the effect of Gyp I on ISO-induced MF and body toxicity in mice, we examined pathological sections of myocardial and liver tissues. Figure 9A shows that the myocardial tissue of the Control group has a clear texture, is consistent in color, and has an orderly arrangement of cells. On the contrary, myocardial tissue in the ISO group showed obvious myocardial interstitial disorder, large-scale inflammatory cell infiltration, and fibrous tissue hyperplasia, accompanied by local tissue necrosis and vacuolar swelling. Notably, Gyp I alleviated the inflammatory response of cardiomyocytes to ISO in a dose-dependent manner and effectively reduced inflammatory damage and the fibrosis area of myocardial tissue. In addition, myocardial tissue of mice in the normal group was yellow in color, and no collagen fibers were seen in the interstitium, while the myocardial tissue of the ISO model group showed a large area of red collagen fibers. In the Gyp I low-, middle-, and high-dose groups, the content of red collagen fibers gradually decreased with an increasing dose (Figure 9B). Thus, the data suggest that Gyp I reduces the content of collagen fibers in myocardial tissue and improves the occurrence of ISO-induced MF.

To determine the effect of Gyp I on ISO-induced cardiac hypertrophy, the cross-sectional area of cardiomyocytes in each group was calculated using Image J software. The cross-sectional area of cardiomyocytes in the normal group was 234.8 ± 7.9 μm^2^, while the cross-sectional area of cardiomyocytes around the inflammatory injury in the ISO group was significantly increased to 577.0 ± 24.3 μm^2^. The cross-sectional areas of peri-inflammatory cells in the low-, middle-, and high-dose groups of Gyp I were 537.4 ± 21.3 μm^2^, 459.7 ± 23.4 μm^2^, and 295.7 ± 31.1 μm^2^, respectively, which were significantly reduced compared with the ISO group (Figure 9C). We also performed H&E staining on liver sections, and the results showed that Gyp I administration alone had no effect on the mouse liver (Figure 9D). Masson staining showed that no inter-tissue collagen fiber hyperplasia was found in the group treated with 40 mg/kg Gyp I alone, which indicated that Gyp I had no toxic effect on liver tissue (Figure 9E).

### 2.10. Effect of Gyp I on IL-1β and IL-18 in the Myocardial Tissue of Mice Induced by ISO

**Figure 10:**
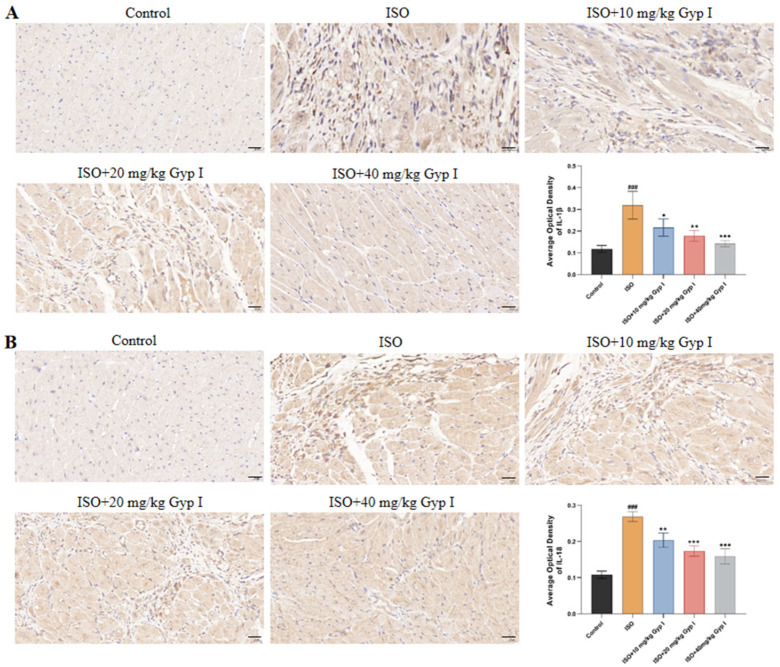
Immunohistochemical images of heart sections: (**A**) IL-1β. (**B**) IL-18 (Scale bar: 20 μm, *n* = 3, mean ± SD, ###: *p* < 0.001 compared with Control group; * *p* < 0.05, **: *p* < 0.01, ***: *p* < 0.001 compared with ISO group).

To further determine that Gyp I prevented ISO-induced fibrosis in mice by reducing the release of inflammatory factors, we performed immunohistochemical staining on myocardial tissue sections using antibodies directed against IL-1β and IL-18. The positive cells in the Control group had no obvious expression, while the cytoplasm of the ISO group showed a large number of brown spots, which represent brown positive expression areas. Compared with the ISO group, the number and degree of positive cells decreased with the increase in Gyp I dosage. By analyzing the average optical density value of the positive cell area, we found that the positive expression of Gyp I to IL-1β in the high-dose group returned to normal. Thus, the results suggest that Gyp I inhibits the release of IL-1β and IL-18 inflammatory factors in the myocardial tissue of ISO-induced mice (Figure 10).

### 2.11. Gyp I Inhibits the TLR4/NF-κB/NLRP3 Pathway In Vivo

**Figure 11:**
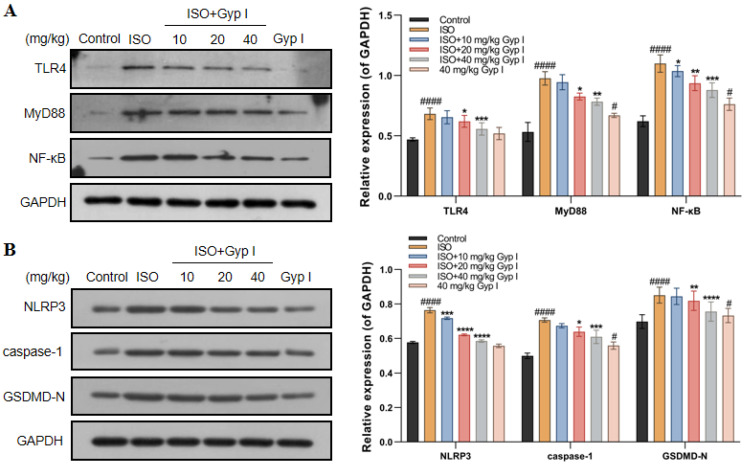
Effect of Gyp I on the expression of the TLR4/NF-κB/NLRP3 signaling pathway in vivo: (**A**) Effects of Gyp I on the expression of TLR4, MyD88, and NF-κB proteins. (**B**) Effects of Gyp I on the expression of NLRP3, caspase-1, and GSDMD-N proteins. Results are presented as mean ± SD of three independent experiments using one-way ANOVA followed by Tukey’s multiple-comparison test (#: *p* < 0.05, ####: *p* < 0.0001 compared with Control group; *: *p* < 0.05, **: *p* < 0.01, ***: *p* < 0.001, ****: *p* < 0.0001 compared with ISO group).

To further explore the mechanism of Gyp I on ISO-induced myocardial tissue in mice, we examined the effect of Gyp I on the TLR4/Myd88/NF-κB signaling pathway by Western blot analysis. The results showed that, after ISO treatment, the protein expression of TLR4, MyD88, and NF-κB was significantly upregulated compared with the Control group (Figure 11A). In addition, NLRP3, caspase-1, and GSDMD-N were significantly increased, as shown in Figure 11B. Notably, compared with the ISO group, Gyp I downregulated the expression of the above proteins in a dose-dependent manner. Furthermore, the Gyp I group treated with a dose of 40 mg/kg alone did not show a significant effect on the expression of the above proteins. These results show that Gyp I blocks the ISO-induced activation of the TLR4/NF-κB/NLRP3 signaling pathway in mice.

### 2.12. Molecular Docking Suggests That Gyp I Could Interact with Several Key Proteins of the TLR4/NF-κB/NLRP3 Signaling Pathway

**Figure 12:**
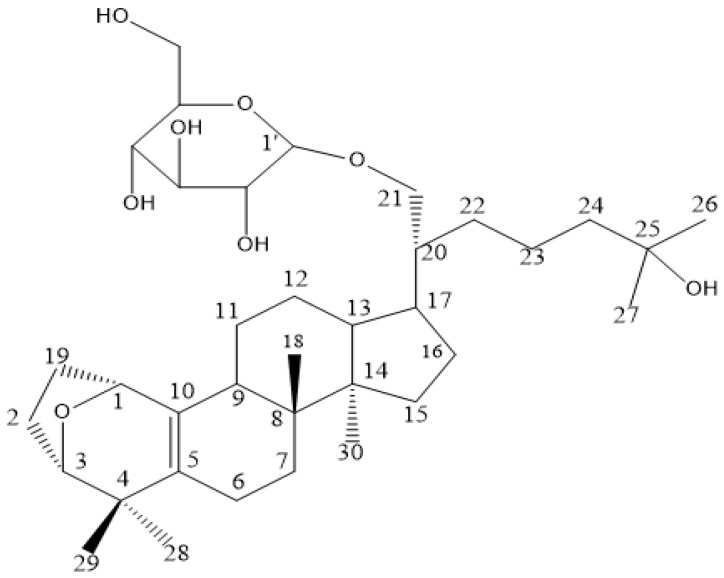
Chemical structure of Gyp I.

**Figure 13:**
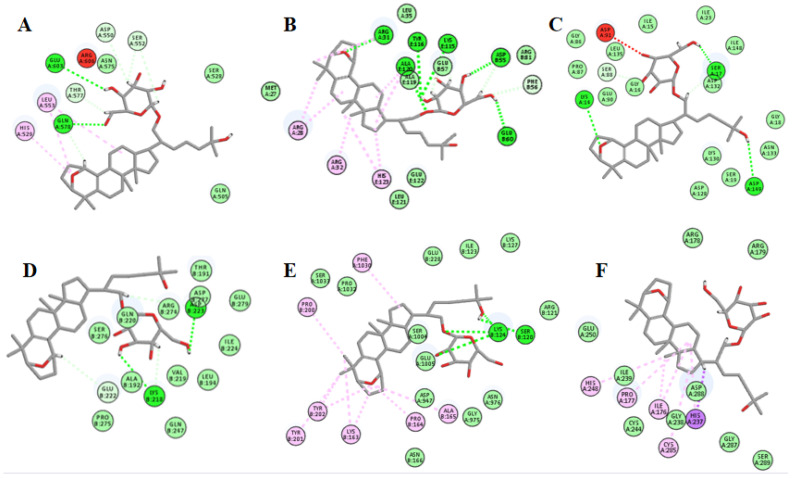
Two-dimensional binding modes showing the interactions between Gyp I and target proteins: (**A**) TLR4. (**B**) MyD88. (**C**) IκBα. (**D**) NF-κB. (**E**) NLRP3. (**F**) caspase-1.

**Figure 14:**
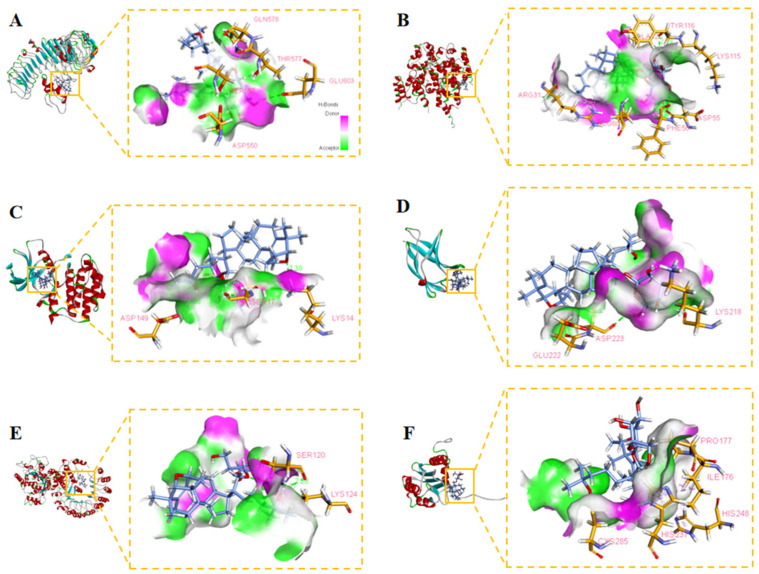
Three-dimensional H-binding modes showing the interactions between Gyp I and target proteins: (**A**) TLR4. (**B**) MyD88. (**C**) IκBα. (**D**) NF-κB. (**E**) NLRP3. (**F**) caspase-1.

The chemical structure of Gyp I is shown in Figure 12. As shown in Figure 13 and Figure 14, and Table 2, we investigated the interactional modes between various key TLR4/NF-κB/NLRP3 signaling pathway proteins and Gyp I, in which the higher the LibDock scores and more abundant form of forces, the stronger the binding ability. The interacting consequences of complex ligand Gyp I-receptor MyD88 were relatively better (Figure 13B and Figure 14B). Specifically, the bridged-ring oxygen atom of Gyp I formed a hydrogen-bonding interaction force with the hydrogen atom on the saturated carbon of ARG31. The oxygen atom of the carbonyl group of ASP55 formed a hydrogen bond with the hydroxyl H on the glycoside of the ligand Gyp I and, at the same time, formed a hydrogen bond with the hydrogen atom of the methylene group of the glycoside side chain. The hydroxyl H of the C’-5 side chain of the ligand glycosyl formed a hydrogen bond interaction with the carbonyl oxygen atom of glutamic acid GLU60. The hydrogen atom of the chiral carbon atom of phenylalanine PHE56 formed a hydrogen bond with the oxygen atom of the hydroxyl group on C’-5. The lysine LYS115 carbonyl oxygen atom formed a hydrogen bond with the hydrogen atom of the alcohol on the glycosyl C’-2 and formed a hydrogen bond interaction with the hydrogen atom attached to C’-2 and C’-3. In addition, the hydrogen atom of C’-2-OH also formed a hydrogen bond with the double-bond oxygen atom of TYR116. The hydrogen atom of the amine group of ALA120 formed a hydrogen bond interaction with the oxygen atom attached to the C-21 position of the compound.

Furthermore, residues ASP550, SER552, THR577, GLN578, and GLU603 interacted with Gyp I and formed hydrogen bonds with the upstream protein TLR4 with a score of 115.271, and we deduce that TLR4 may be an essential target protein (Figure 13A and Figure 14A). Further, the LibDock score of IκBα was 113.576, and it displayed four hydrogen bonds involving LYS14, SER17, SER88, and ASP149 (Figure 13C and Figure 14C). Moreover, residues LYS218, GLU222, and ASP223 interacted with Gyp I and formed hydrogen bonds with the downstream protein NF-κB with a score of 121.299 (Figure 13D and Figure 14D); residues SER120 and LYS124 of NLRP3 formed hydrogen bonds with a score of 110.726 (Figure 13E and Figure 14E); and residues ARG178, GLY287, and ASP288 of caspase-1 formed hydrogen bonds with a score of 116.55 (Figure 13F and Figure 14F).

## 3. Discussion

In this study, we showed in vitro that Gyp I alleviated H9c2 cell injury and protected ISO-induced myocardial damage. Furthermore, Gyp I ameliorated ISO-induced MF by attenuating oxidative stress, inflammation, and hypertrophy through the inhibition of the TLR4/NF-κB/NLRP3 signaling pathway.

In the pathological state, MF proliferates and transforms into myofibroblasts, thereby increasing ventricular stiffness and reducing myocardial diastolic and systolic function, accompanied by the deposition of massive collagen fibers in the extracellular matrix [39,40]. Gyp I is a triterpenoid secondary saponin obtained from the acid hydrolysis of total saponins from Jiaogulan. The present study indicates that Gyp I had little cytotoxicity on H9c2 cells when used at a dose of 0–15 μM. Similar results were shown in vivo, where the Gyp I alone intervention group (40 mg/kg) did not show an effect on the gross morphology of the heart and liver, and the pathological condition of the liver sections observed by H&E and Masson staining was good. We speculate that Gyp I is a potentially potent compound for preventing myocardial damage.

ISO, a synthetic nonselective β-adrenergic receptor agonist, has been shown to induce myocardial fibroblasts [41]. The current study showed that, after treating H9c2 cells with ISO (200 μM) for 48 h, cardiomyocyte apoptosis reached the best model level in vitro. However, long-term drug stimulation takes the risk of impairing cardiac contractility and pumping function, and dose control also determines the morbidity of the animals [42]. Under normal physiological conditions, the weight and volume of the heart and left ventricle increased proportionally with the growth of the body, thereby maintaining the HWI-to-LVWI ratio within a constant range [43]. Continuous subcutaneous injection of ISO (10 mg/kg) in mice for 21 days induced a model of MF, while Gyp I (10, 20, and 40 mg/kg) dose-dependently improved HWI and LVWI indices and restored normal cardiac morphology in mice. Measuring cardiac electrical activity though ECG modalities, Gyp I improved both myocardial contractility and the pumping function in ISO-induced mice.

Previous studies have shown that patients with cardiovascular disease not only exhibit ventricular systolic dysfunction but also are often associated with the main features of pathological myocardial fibrosis, cardiac hypertrophy, and re-expression of atrial natriuretic peptide and B-type natriuretic peptide [13,44,45]. We further examined the pathological features of myocardial tissue and found that Gyp I ameliorated myocardial interstitial disorder in ISO-induced mice, reduced inflammatory cell infiltration in myocardial tissue, and moderated the deposition of collagen fibers in myocardial tissue. Furthermore, we observed an increase in the cross-sectional area of inflammatory cardiomyocytes after ISO induction and examined the mRNA expression of embryonic gene markers ANP and BNP. The results showed that Gyp I significantly reduced the level of cardiac hypertrophy while intervening in MF.

Cardiac injury caused by ISO results in cardiomyocyte necrosis, massive cell lysis, and the excretion of cardiac biomarker enzymes into the blood circulation. We detected a reduction in levels of LDH and CK in a dose-dependent manner after Gyp I intervention. Moreover, ISO-induced mice had altered levels of oxidative stress that promoted inflammatory signaling and compromised the normal heart function. Various antioxidant enzymes, such as SOD, CAT, and GSH-Px, constitute the defense system against free radical oxidative damage in the body and are of great significance for maintaining the balance of oxidation/antioxidation in the body [46]. The levels of three antioxidant enzymes and MDA in tissues were detected, and it was found that the Gyp I intervention group showed a decreased level of MDA and an increased content of SOD, CAT, and GSH-Px compared with the ISO group in a dose-dependent manner.

Apoptosis is a routine type of programmed death and an important cause of ISO-induced myocardial injury [47,48]. Flow cytometry showed that Gyp I could significantly reduce the apoptosis rate of damaged cells. Moreover, Western blot analysis showed that Gyp I upregulated the ratio of Bcl-2/Bax in a dose-dependent manner, promoted the inactivation of Procaspase-3, and reduced the expression level of Cleaved caspase-3. However, the inhibition of Cleaved caspase-3 by Gyp I cannot further cleave the downstream substrate PARP-1 involved in the apoptosis process, thereby hindering the expression of Cleaved PARP-1 and inhibiting the occurrence of apoptosis.

Inflammation is generally closely associated with fibrosis [49]. Following an inflammatory response in cardiac tissue affected by chronic ISO treatment, a substantial secretion of the essential inflammation-related cytokines IL-1β and IL-18 is processed and delivered to the heart during cardiac stress, due to the NLPR3 inflammasome-associated cytokines being activated upon stimulation [34,50]. In a recent study, it was demonstrated that inflammasome activation plays an important role in ISO-induced MF [30]. Using immunohistochemistry to detect the expression levels of IL-18 and IL-1β in tissues, it was demonstrated that ISO induced an inflammatory response and released inflammatory factors, while Gyp I intervention improved myocardial inflammation in mice with MF.

It has been reported that cardiomyocytes, in addition to undergoing apoptosis, also trigger an inflammatory cascade through the TLR4/NF-κB signaling pathway after ISO injury [51,52]. Inhibition of the NLRP3 inflammasome can effectively reduce the ISO-induced inflammatory response [53]. TLR4 is widely present in many cardiovascular diseases. It induces the nuclear expression of NF-κB by triggering inflammatory signaling cascades and specifically binds to a variety of proinflammatory cytokines to induce MF [25]. We preliminarily docked Gyp I with six receptor proteins, including TLR4, MyD88, NF-κB, NLRP3, caspase-1, and GSDMD-N, and they all showed different degrees of hydrogen-bonding force and various forms of hydrophobic interaction. The abovementioned target proteins were further verified by Western blot analysis, and the results showed that Gyp I could downregulate the expression levels of TLR4 and other target proteins in the TLR4/NF-κB/NLRP3 signaling pathway in vitro and in vivo.

In addition, our current study only verified that Gyp I has a protective effect on MF, but its bioavailability and pharmacokinetics are the directions of future research. The large-scale preparation of Gyp I is also the focus of future research.

## 4. Materials and Methods

### 4.1. Sample

Saponins from *G. pentaphyllum* (>80%) were purchased from Hunan Province Jiuhui Modern Chinese Materia Medica Co., Ltd. and were hydrolyzed with a hydrochloric acid aqueous solution ratio of 3:2. The hydrolysates were subjected to silica gel column chromatography, using a stepwise system of CH_2_Cl_2_/MeOH (100:0~0:100, *v*/*v*), coupled with high-performance liquid chromatography to afford Gyp I (a white amorphous solid with a purity of up to 98.5%).

### 4.2. Chemical Reagents and Antibody

Fetal bovine serum (FBS) was purchased from Thermo Fisher Scientific (Shanghai, China). ISO was procured from Sigma Industry Co., Ltd. (St Louis, MO, USA). In addition, dual anti-sterilization solution (penicillin/streptomycin solution), 0.25% trypsin-EDTA, phosphate-buffered saline (PBS), and Dulbecco’s modified Eagle’s medium (DMEM) were all purchased from Meilun Biotech (Dalian, China). MTT (3-(4, 5-dimethylthiazol-2-yl)-2, 5-diphenyltetrazolium bromide) was purchased from Beyotime Biotechnology (Shanghai, China). Dimethyl sulfoxide (DMSO) was purchased from Meilun Biotech (Dalian, China).

Antibody against β-actin was purchased from Servicebio (Wuhan, China), and the caspase-3, caspase-1, PARP, and cleaved PARP antibodies were obtained from Biosystems Dogs (Shenyang, China). Other antibodies were all purchased from Beyotime Biotechnology (Shanghai, China).

### 4.3. Cell Culture

The H9c2 rat cardiomyocytes, purchased from Meilun Biotechnology Company (Suzhou, China), were cultured in Dulbecco’s modified Eagle’s medium (DMEM) supplemented with 10% (*v*/*v*) fetal bovine serum and 1% (*v*/*v*) dual anti-sterilization solution at 37 °C in a humidified atmosphere filled with 5% CO_2_.

### 4.4. Cell Viability Assay

Cardiomyocytes were plated in a 96-well plate (5 × 104 cells/well) and incubated for 24 h. Gyp I was dissolved in cell-grade DMSO and prepared as a mother liquor with a final concentration of 1 × 104 μM in advance. After the cells were completely attached, Gyp I was diluted in a gradient of 2.5, 5, 7.5, 10, 12.5, and 15 μM, and the cell viability was detected for 24 h. MTT was added to each well, and the cells were further incubated for 4 h and then mixed with DMSO sequentially. A microplate reader was used to detect the absorbance of each well at 490 nm to calculate cell viability. In addition, different concentrations (50–300 μM) of ISO induced H9c2 for 24 and 48 h to construct myocardial injury models.

To examine the effect of Gyp I on ISO-induced cardiotoxicity of H9c2 cells, Gyp I (2.5–15 μM) protection was performed for 4 h before ISO (200 μM) treatment and Gyp I co-incubation for 48 h. Subsequently, MTT assays were performed as previously described.

### 4.5. Annexin V/Propidium Iodide (PI) Assay

H9c2 cells (1 × 105 cells/well) were seeded in 6-well plates and cultured for 24 h. When the cells were in good condition and in the logarithmic growth phase, Gyp I was added at doses of 5, 7.5, and 10 μM for 4 h to pre-protect cells. Then, each concentration of Gyp I and ISO (200 μM) was co-incubated for 48 h. When the cells were harvested, they were centrifuged at 1200 rpm for 5 min and washed with PBS. In the lase, the cells were placed in binding buffer (400 μL) mixed with Annexin V- FITC (4 μL) and PI solution (4 μL) in a dark environment. Flow cytometry was used to measure cell apoptosis.

### 4.6. Experimental Animals and Groups

Six-week-old male C57BL/6 mice weighing 18–20 g were purchased from Changsheng Bioscience Co., Ltd. (Liaoning, China), and routine experiments were performed in accordance with the “Regulations on the Management of Laboratory Animals” issued by the National Science and Technology Commission. Animal treatments were given official approval by the Institutional Animal Care and Use Committee (permission number: SYXK (Liao) 2021–0009) of Shenyang Pharmaceutical University. Experiments were performed at a temperature of 20–22 °C and a humidity of 40–60%; a 12 h light/dark cycle was used for feeding, during which specific-pathogen-free (SPF)-grade sterile water and feed were used.

A total of 48 mice were randomly assigned to Control (vehicle, p.o.), ISO (10 mg/kg, i.h.), and ISO (10 mg/kg, i.h.) + Gyp I intervention groups (10, 20, and 40 mg/kg, p.o.) with 8 mice per group for 21 days. At the end of treatment, mice were euthanized, and their hearts were harvested and weighed for calculating heart-to-weight ratio before biochemical indicators, RT-PCR, and Western blot analyses were performed. Liver and heart tissues were harvested for histopathology analysis. In addition, serum was used to measure LDH and serum CK indicators.

### 4.7. Analysis of HWI and LVWI

The body weight of mice was recorded every three days during the feeding process. All mice were made to fast for 12 h after the last administration and sacrificed by decapitation; their hearts were rapidly dissected, and excess blood vessels and tissues were removed. The hearts were immersed and washed in 0.9% normal saline, the excess water was removed with filter paper, and the weight of the heart was recorded. Then, the left ventricle was stripped and weighed, and the HWI and LVWI were calculated, respectively.

### 4.8. ECG Analysis

One day before sacrifice, mice were anesthetized with chloral hydrate by intraperitoneal injection, placed on a fixation plate, and limbs were spread. Two needle electrodes were attached to the mouse’s forelimb, and one needle electrode was attached to the mouse’s left hind limb. Using the Philips HeartStart XL monitor/defibrillator machine, ECG readings were performed, and ST-wave changes from the ECG were analyzed.

### 4.9. Serum Biomarker Analysis

Blood from mice was collected, and serum was centrifuged (3000 rpm for 15 min) before the expression levels of various biochemical indicators (LDH and CK) were detected using an Automatic biochemical analyzer Chemray 800.

### 4.10. Heart Oxidative Stress Markers

Oxidative stress was measured in myocardial tissue homogenate as malondialdehyde (MDA), glutathione (GSH-Px), catalase (CAT), and superoxide dismutase (SOD) according to the kit (Nanjing Jiancheng Biological Company, China) method for evaluation.

### 4.11. Real-Time Quantitative RT-PCR

For the total RNA extraction of tissues, a small portion of heart tissue samples was removed, frozen in liquid nitrogen, and homogenized in the reagent described above with 5 mm stainless steel beads according to the manufacturer’s specifications (Servicebio). Servicebio^®^RT First Strand cDNA Synthesis Kit was used to reverse transcribe RNA into cDNA. RT-PCR was performed using a LightCycle^®^ 480 II fluorescence quantitative PCR instrument (Roche, Switzerland) after detecting the reaction with a 2 × SYBR Green qPCR Master Mix (None ROX). The expression level of mRNAs was normalized to the GAPDH gene, and relative gene expression changes were calculated using the 2^−ΔΔCt^ method. The *t* test was used to assess differences in expression. The primer sequences (Table 2) were designed using Primer 5.0.

### 4.12. H&E Straining

The tissues were fixed in 10% paraformaldehyde solution, embedded in paraffin, cut into 5 μm slices, deparaffinized with xylene, soaked in a mixture of xylene and pure alcohol (1:1) for 5 min, immersed in gradient alcohol after 5 min, and dyed with hematoxylin for 2–5 min. Next, the color was separated with 1% hydrochloric acid alcohol for a few seconds, after which the sections were rinsed with tap water and distilled water 3 times, eosin staining was performed for 2–5 min, and the sections were dehydrated with gradient alcohol. Finally, the cells were observed, and pictures were taken under a microscope.

### 4.13. Sirius Red Staining

After Sirius red staining for 1–1.5 h, the staining solution on the surface of the sections was rinsed with running water, and nuclei were counterstained with hematoxylin for 1 min. After differentiation and reverse blue operations, the cells were washed with running water. Sections were routinely dehydrated until transparent and sealed with neutral resin. Finally, images were taken under an optical microscope for observation.

### 4.14. Masson Straining

After dewaxing to water, the sections were co-stained with hematoxylin and ferric chloride aqueous solution for 10 min, differentiated with acidic ethanol for 10 s, and washed with water. After returning to blue with an aqueous ammonia solution, the sections were washed with water and rinsed with distilled water. Afterwards, Ponceau Fuchsin was stained with droplets for 10 min, treated with aqueous acetic acid and aqueous phosphomolybdic acid, washed with aqueous acetic acid, counterstained with aniline blue, etc., dehydrated until transparent, and sealed with neutral resin.

### 4.15. Immunohistochemistry

The sections were immersed in Tris/EDTA buffer and heated in the microwave for 15 min. After antigen retrieval, the sections were incubated with 3% hydrogen peroxide for 10 min to block endogenous peroxidase activity. After peroxidase inactivation, the sections were blocked with 3% BSA for 30 min at room temperature. After incubating the sections with primary antibody at 4 °C overnight, they were washed 3 times with TBST, incubated with the corresponding HRP-conjugated secondary antibody for 50 min at room temperature in the dark, and washed 3 times with TBST; DAB formed a brown precipitate at the antigenic site. Sections were counterstained with hematoxylin, and the slides were dried, mounted with neutral glue, and observed under an optical microscope.

### 4.16. Western Blot Analysis

H9c2 cells were lysed on ice with RIPA buffer (Dalian Meilun, Dalian, China) containing protease inhibitors (PMSF) (Dalian Meilun, Dalian, China) and phosphatase inhibitors (sodium fluoride and Na3VO4) (Wuhan Servicebio, Hubei, China) for 30 min. The supernatant was then centrifuged to obtain the total protein extract. A BCA kit (Shanghai Beyotime Biotechnology, Shanghai, China) was used to detect the standard protein concentration, and SDS loading buffer protein was added to quantify the protein concentrations of all samples. After a series of heating and boiling procedures, the samples were finally stored at −20 °C for later use. For the protein extraction of tissues, a small piece of heart tissue was removed, frozen in liquid nitrogen, and homogenized in the lysis buffer described above with 5 mm stainless steel beads in the grinder (Wuhan Servicebio, China). The remaining operations were then performed as described above.

The protein specimens were separated by SDS-PAGE (8% and 12% gels) and transferred to the nitrocellulose filter (NC) membrane before blocking with 5% skimmed milk for 1 h. Subsequently, the primary antibodies were incubated overnight at 4 °C and washed with TBST. The bands were incubated with goat anti-rabbit/mouse antibodies (1:1000) (Shanghai Beyotime Biotechnology, Shanghai, China) labeled with horseradish peroxidase for 1 h. After the washing procedure was completed, protein expression was displayed on the film by preparing and adding ECL luminescent liquid (Wuhan Servicebio, Hubei, China).

### 4.17. Molecular Docking

A molecular docking study was performed with the Discovery Studio (DS) 2016/LibDock protocol to explore the binding modes of Gyp I with the proteins of the TLR4/NF-κB/NLRP3 signaling pathway. The 2D chemical structures of small molecules were drawn by Chemdraw software and then transferred to DS to produce 512 stereoisomers. In addition, all protein crystal structures were deposited into the PDB databank, and the IDs are listed in Table 1. The measure of interaction between Gyp I and target proteins was determined by calculating the LibDock score.

### 4.18. Statistical Analysis

All values are expressed as mean ± standard deviation (SD) from three or five repeats (*n* = 3 or *n* = 5) of each experiment performed in triplicates. Student’s *t* test was used to perform statistical analyses of two groups, and one-way ANVOA was used for more than two groups. * *p* < 0.05, ** *p* < 0.01, *** *p* < 0.001, and **** *p* < 0.0001 were considered to indicate significant differences.

## 5. Conclusions

In this study, we showed in vitro that Gyp I alleviated H9c2 cell injury and protected ISO-induced myocardial damage. Furthermore, Gyp I ameliorated ISO-induced MF by attenuating oxidative stress, inflammation, and hypertrophy through the inhibition of the TLR4/NF-κB/NLRP3 signaling pathway in vivo. In view of its cardioprotective and nontoxic properties, Gyp I has the potential to be a novel and effective drug to treat MF.

## Figures and Tables

**Table 1:** Primer sequences for hypertrophy genes.

Primer Name		Primer Sequence (5′-3′)	Fragment Length (bp)
ANP	forward	CTTCTTCCTCGTCTTGGCCTTT	114
	reverse	TCCAGGTGGTCTAGCAGGTTCT	114
BNP	forward	GCTGCTGGAGCTGATAAGAGAA	194
	reverse	CGATCCGGTCTATCTTGTGCC	194
GAPDH	forward	CCTCGTCCCGTAGACAAAATG	133
	reverse	TGAGGTCAATGAAGGGGTCGT	133

**Table 2:** Interactions between Gyp I and target proteins.

Entry	Protein	PDB ID	LibDock Score	Interaction
1	TLR4	3FXI	115.271	H bonds: GLN578, GLU603 Interacting residues: ASP550, HIS529, SER552, LEU553, THR577
2	MyD88	3MOP	129.773	H bonds: ARE31, ASP55, GLU57, ALU60, LYS115, TYR116, ALA119, ALA120 Interacting residues: ARG28, ARG31, ARG32, PHE56, ALA120, HIS123
3	I-κBα	4KBA	113.576	H bonds: LYS14, SER17, ASP132, ASP149 Interacting residues: SER88
4	NF-κB	1MY5	121.299	H bonds: LYS218, ASP223, ARG274 Interacting residues: GLU222
5	NLRP3	6NPY	110.726	H bonds: SER120, LYS124 Interacting residues: LYS163, PRO164, ALA165, PRO200, TYR201, TYR202, PHE1030
6	caspase-1	1RWX	116.82	Interacting residues: ILE176, PRO177, HIS237, HIS248, CYS285

## Data Availability

The data that support the findings of this study are available from the corresponding author upon reasonable request.
